# Longitudinal Relationships Among Fear of COVID-19, Smartphone Online Self-Disclosure, Happiness, and Psychological Well-being: Survey Study

**DOI:** 10.2196/28700

**Published:** 2021-09-27

**Authors:** Jörg Matthes, Kevin Koban, Ariadne Neureiter, Anja Stevic

**Affiliations:** 1 Department of Communication University of Vienna Vienna Austria

**Keywords:** COVID-19 pandemic, fear, self-disclosure, happiness, well-being, panel study, smartphones, online platform, social media

## Abstract

**Background:**

Given that governmental prevention measures restricted most face-to-face communications, online self-disclosure via smartphones emerged as an alternative coping strategy that aimed at reducing the impact of the COVID-19 pandemic on people’s psychological health. Prepandemic research demonstrated that online self-disclosure benefits people’s psychological health by establishing meaningful relationships, obtaining social support, and achieving self-acceptance, particularly in times of crisis. However, it is unclear whether these dynamics transition well to lockdown conditions where online self-disclosure must stand almost entirely on its own. Longitudinal investigations are needed to gain insights into the psychological functionalities of online self-disclosure during the COVID-19 pandemic.

**Objective:**

This study aimed to determine the temporal associations between smartphone online self-disclosure (as a communicative behavior) and critical indicators of psychological health (including psychopathological, as well as hedonic and eudaimonic states) during the first COVID-19 lockdown in Austria.

**Methods:**

We conducted a representative 2-wave panel survey between late March/April 2020 and May 2020. A total of 416 participants completed both waves (43.1% attrition rate, given n=731 participants who completed the first wave). A partially metric measurement invariant overtime structural equation model was used to determine the temporal associations among online self-disclosure, fear of COVID-19, happiness, and psychological well-being.

**Results:**

The analysis revealed that fear of COVID-19 significantly predicted online self-disclosure over time (*b*=0.24, *P*=.003) and happiness over time (*b*=−0.14, *P*=.04), but not psychological well-being (*b*=0.03, *P*=.48), that is, stronger COVID-19 fears at T1 prompted more online self-disclosure and less happiness at T2. Online self-disclosure, on the other hand, significantly predicted happiness (*b*=0.09, *P*=.02), but neither fear of COVID-19 (*b*=−0.01, *P*=.57) nor psychological well-being (*b*=−0.01, *P*=.57) over time. Participants who engaged more strongly in online self-disclosure at T1 felt happier at T2, but they did not differ from less-disclosing participants concerning COVID-19 fears and psychological well-being at T2. Importantly, happiness and psychological well-being were significantly related over time (happiness T1 → psychological well-being T2: *b*=0.11, *P*<.001; psychological well-being T1 → happiness T2: *b*=0.42, *P*<.001).

**Conclusions:**

Our findings suggest that online self-disclosure might play a pivotal role in coping with pandemic stressors. With restrictions on their options, individuals increasingly turn to their smartphones and social media to disclose their feelings, problems, and concerns during lockdown. While online self-disclosure might not alleviate fears or improve psychological well-being, our results demonstrate that it made people experience more happiness during this crisis. This psychological resource may help them withstand the severe psychological consequences of the COVID-19 crisis over longer timeframes.

## Introduction

### Background

Across the world, fears (ie, an intense emotion emerging from a perceived imminent threat [1]) related to the COVID-19 pandemic are having a pervasive influence on people’s psychological health (understood in a “balanced” way as a continuum that includes both the *absence* of psychopathological and the *presence* of positive psychological states [2]) that extends beyond its immediate threat to physical health [3] and warrants extensive research efforts to face current and future challenges [4]. With respect to these COVID-19 fears, Schimmenti et al [5] used the apocalyptical label of the “four horsemen” to emphasize how significant various states of fear are in explaining people’s behavior during the pandemic. According to them, COVID-19 fear involves the following four domains that are mutually connected: fear of/for the body (ie, the body as a potential source of danger and hypervigilance), fear of/for significant others (ie, interpersonal relationships as a potential threat and source for sorrow), fear of (not) knowing (ie, information as a potentially unsettling necessity), and fear of (in)action (ie, decisions that once made or avoided can be potentially harmful). Empirical evidence broadly supports their emphasis showing enhanced levels of fear and other harmful emotional states (such as depressive tendencies and stress) during the COVID-19 pandemic [6-9].

In addition to the fear of getting infected, most governments issued prevention orders that minimized public life and called for physical distance as the appropriate interpersonal norm for private and public gatherings. Consequently, unrestricted face-to-face social environments disappeared almost completely. To deal with this profound change and related adverse psychosocial states, individuals exercised various coping strategies [10,11].

This study focuses on one key coping strategy, *online self-disclosure.* Self-disclosure is defined as the communication of self-related private information to other people [1]. Self-disclosure has long been established as a critical coping behavior in institutionalized therapeutic contexts [12] and informal private settings [13]. In times of the pandemic and particularly during lockdowns, self-disclosure may instead be exercised frequently on social media, most notably via mobile devices such as smartphones. Affordances in such online contexts differ from most offline contexts when it comes to the frequency, breadth (ie, diversity), and depth (ie, intimacy) of self-disclosures [14,15]. Yet, prior research suggests that online self-disclosure can directly contribute to people’s psychological health (as an efficient coping behavior) and indirectly by prompting offline self-disclosure as an additional coping resource [16].

Against this background, this study looks at the consequences of online self-disclosures during the first lockdown period of the COVID-19 pandemic in 2020.

This study contributes to our understanding of digital media’s roles in times of a pandemic in several ways. First, we, for the first time, shed light on how self-disclosure via smartphones can help individuals cope with pandemic-related stressors, ultimately fostering psychological health. Second, and related to that, we focus on people’s online self-disclosure as a communicative *behavior* rather than on *experiential* constructs such as perceived social support and feelings of loneliness. Third, from a methodological standpoint, this study contrasts itself from the great abundance of cross-sectional research by reporting results from a 2-wave longitudinal survey that took place during the first COVID-19 lockdown in Austria. Our sample followed representative quota data, allowing for greater generalizability of the results. Moreover, by employing measurement invariant overtime structural equation models, we can provide the first glimpse into temporal order and directional associations between key constructs. Overall, our study aims at providing the first empirical evidence about how online self-disclosure in times of COVID-19 serves as an effective coping behavior that is provoked by COVID-related fears and leads to improved psychological health.

### Fear of COVID-19 as a Predictor of Online Self-Disclosure, Happiness, and Psychological Well-being

In comparison to previous years, overall media use has been on the rise during the COVID-19 pandemic [17,18]. Interestingly, a study by Ohme et al [19] revealed that people tend to prioritize mobile messengers over more public social media platforms when confronted with pandemic-related developments, indicating an immediate need for social support. Large-scale analyses of people’s public social media activities exhibited more verbal expressions that would typically be considered as symptomatic of mental health hazards (eg, anxiety, depression, stress, and suicidal ideation), and outreaches for emotional and informational support [20]. Such private and public media activities align with the basic premise of the fever model of disclosure [21]. According to this theoretical approach, self-disclosure is understood as a self-regulative behavior that emerges as a function of experienced distress and aims for maintaining psychological health. Self-disclosing communications are assumed to become more likely under elevated levels of distress to elicit support from close others that may help people cope.

Undoubtedly, the COVID-19 pandemic has been a significant stressor for individuals, especially with respect to fear, as people are “worried, fearful, and uncertain about COVID-19 and the consequences it will have for themselves, their families, communities, and the nation” [6]. Following the fever model of disclosure [21], we theorize that online self-disclosure is one possible coping strategy that individuals use to deal with these fears during lockdown. Following this argumentation, we expected that COVID-19 fears lead to more frequent online self-disclosures. The first hypothesis (H1) was as follows: Fear of COVID-19 positively predicts online self-disclosure on social media over time.

Except occasionally for playful media-induced feelings [22], fear is generally regarded as a negatively experienced emotion with high valence and arousal [23] that, if not reappraised or extinguished, can damage people’s psychological health [24,25]. Protection measures (such as intensified online self-disclosure) may not always suffice to reappraise or eliminate the complex web of fears. In the case of the COVID-19 pandemic (in particular, during the first lockdown), people have been continuously afraid of and hypervigilant about any yet unknown or newly arising threats [5]. It is, therefore, not surprising that findings point toward a negative link between people’s COVID-19 fears and their psychological health [26]. Since there were no major alleviating events during the timeframe of this study (ie, March/April to May 2020) that may have mitigated its impact, we hypothesized that people’s COVID-19–related fear will have a negative influence on the following two key indicators of psychological health: happiness and psychological well-being. These indicators were selected because each reflects one of the following two dominant traditions in positive psychology: hedonism and eudaimonism [27,28]. More specifically, we follow a previous report [29] in their definition of *happiness* as the presence of pleasant and the absence of unpleasant emotions, and their definition of *psychological well-being* as an umbrella term that includes various eudaimonic experiences, such as meaning and purpose in life, satisfied psychological needs, and self-acceptance. While both indicators contribute individually to psychological health, it is their interaction that can lead people to flourish [30,31]. Due to this mutually reinforcing role in promoting the proverbial good life, we examined both their relationships with fear over time. The second hypothesis (H2) was as follows: Fear of COVID-19 negatively predicts (1) happiness and (2) psychological well-being over time.

### Online Self-Disclosure as a Predictor of Happiness, Psychological Well-being, and Fear of COVID-19

Everything considered, prepandemic research suggests that online self-disclosure can have positive consequences for psychological health. Luo and Hancock [32] detailed different mechanisms through which people may benefit. Most notably, disclosing personal feelings to others in social media constitutes the basis for meaningful online conversations that promote social capital [33] and interpersonal relatedness [34], and help against loneliness [35] and stressful life events [36]. As evidenced in the study by Trepte et al [16], online self-disclosure may also provide a fertile ground for long-term impacts, since it reinforces online self-disclosure in the course of time and carries over to offline (ie, face-to-face) self-disclosures.

This advantageousness might be reinforced during COVID-19 as people may have become more aware and intentional of their online self-disclosures [37]. Within formal therapeutic settings, research has highlighted how online treatments during the pandemic can facilitate self-disclosures and their benefits for psychological health [38,39]. More informally, the unique affordances of social media have been argued to facilitate self-disclosures during COVID-19 that, in turn, may help people to maintain a sense of control [40]. Nevertheless, Canale et al [41] did not find a significant direct correlation between emotional online self-disclosure and psychological health during COVID-19. Indirect effects, however, align with prepandemic links between online self-disclosure and social support. Here, cross-sectional studies have validated the positive effect of social support, very likely following online self-disclosure, on individuals’ psychological health [41,42]. In other words, individuals who disclosed their COVID-19–related concerns online were more likely to receive immediate social support, such that they understood or got the impression that they are not alone. Based on these findings, we assumed that online self-disclosure should be positively related to happiness and psychological well-being during the pandemic. The third hypothesis (H3) was as follows: Online self-disclosure on social media positively predicts (1) happiness and (2) psychological well-being over time.

**Figure 1:**
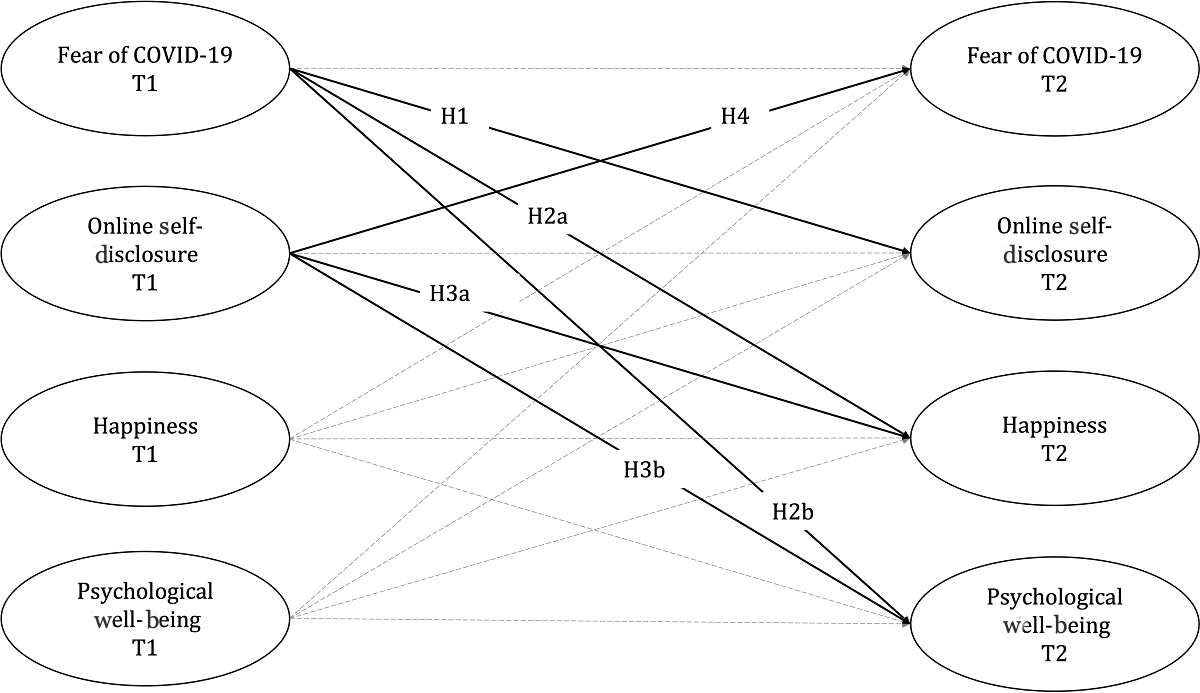
Full theoretical model of this study. Solid lines indicate the paths that were included in the hypotheses, and dashed lines indicate the paths that were involved in statistical analysis.

Online self-disclosure during the pandemic may help to not only improve positive psychological states at large but also cope with adverse feelings. The first cross-sectional evidence supports this position. As presented in the study by Canale et al [41], expressing emotions deliberately in online conversations was found to be beneficially associated with coping with COVID-19–related trauma. In this context, emotional online self-disclosures may be understood as a behavioral manifestation linked to adaptive emotion regulation strategies (such as acceptance and perspectivization) that help people cope with their COVID fears [43]. However, cross-sectional investigations cannot determine the directions in which these associations may work. Participants in the study by Canale et al [41] may have overcome COVID-19 trauma due to stronger self-disclosure activities; yet, considering that online conversations are often biased toward displaying positive information [44], it is equally possible that those participants who had scored lower on COVID-19 trauma were merely more motivated to disclose their feelings online. Still, following the report by Luo and Hancock [32], we assumed that people who engage in online self-disclosure during the pandemic might experience less fear about it at a later point in time. The fourth hypothesis (H4) was as follows: Online self-disclosure on social media negatively predicts fear of COVID-19 over time. The full theoretical model is shown in Figure 1.

## Methods

### Data Statement

The data of this paper (which is available at Open Science Framework Project Space [45]) is part of a more comprehensive project examining longitudinal relationships between smartphone use in times of COVID-19 and well-being. Importantly, the paper only reports variables that are relevant to the present research interest.

### Sampling and Procedure

We conducted a 2-wave panel survey during the first COVID-19 lockdown in Austria. Data collection took place in late March/early April 2020 (T1) and May 2020 (T2) within a 1-month interval. This 1-month (ie, on average) interval was chosen because we were looking at medium-term processes unfolding over several weeks. There was a strong and unpredictable dynamic during the early stages of the COVID-19 pandemic. A larger time span (eg, 6 or 12 months) may have been problematic because several confounding events could have occurred during data collection, making the findings of a larger time-frame meaningless.

Before we started collecting data, we sought ethical clearance from the Institutional Review Board of the Department of Communication at the University of Vienna. Participants were recruited by the professional online polling institute Dynata. Complementarily, another sample was collected simultaneously at the University of Vienna using the same questionnaire, methodology, and quota plan. We used representative quotas for age, gender, and educational level in Austria. For participants to take part in the data collection, they were required to provide their consent, report whether they are using a smartphone, and be at least 16 years of age.

A total of 731 participants (mean age 40.49 years, SD 13.33 years; 394 [53.9%] women; 150 [20.5%] with lower secondary education or less, 340 [46.5%] with vocational school education or secondary education, and 241 [33.0%] with complete university education) completed the survey in the first wave. Among these participants, 416 also completed the second wave (mean age 41.97 years, SD 13.59 years; 226 [54.3%] women; 90 [21.6%] with lower secondary education or less, 187 [45.0%] with vocational school education or secondary education, and 139 [33.4%] with complete university education). The attrition rate between the two waves was 43.1%. There was no significant difference between participants who dropped out after the first wave (T1; n=315) and participants who also completed the second wave (T2; n=416) regarding gender (*χ*^2^_2_=1.37, *P*=.50, Cramér *V*=0.043), education (*χ*^2^_5_=5.33, *P*=.38, *V*=0.085), fear of COVID-19 (*t*_729_=0.50, *P*=.62, Cohen *d*=0.037), online self-disclosure on social media (*t*_729_=−1.45, *P*=.15, *d*=−0.108), happiness (*t*_729_=0.40, *P*=.69, *d*=0.030), and psychological well-being (*t*_631.01_=0.42, *P*=.68, *d*=0.032). Participants who dropped out at T2 had a lower age (mean 38.53 years, SD 12.80 years) than participants who participated in both surveys (mean 41.98 years, SD 13.55 years; *t*_729_=3.49, *P*=.001, *d*=0.261).

### Measures

All implemented measures were based on established scales. Given that this study was part of a more comprehensive survey, we opted for short scales that were constructed using high-loading items of these established scales to reduce dropout and prevent respondent fatigue while at the same time minimize reliability and validity losses.

#### Fear of COVID-19

Similar to Ahorsu et al [8], we employed 2 items measuring fear of COVID-19. We asked participants to indicate their agreement to the following 2 statements: “The coronavirus scares me” and “I am afraid that I could get infected with the coronavirus.” Participants answered on a 5-point Likert scale ranging from “strongly disagree” to “strongly agree” (T1: Cronbach *α*=.76; mean 2.77, SD 1.07; T2: *α*=.80; mean 2.42, SD 1.07). Principal component analysis resulted in a 1-dimensional scale (eigenvalue=1.61; accounting for 80.03% of the variance).

#### Online Self-Disclosure on Social Media

We measured online self-disclosure on social media, with 4 items adapted from Schouten et al [35,46]. We asked participants how often they have used their smartphones during the last week to communicate the following content on social media platforms: “personal feelings about the corona crisis,” “concerns about the novel coronavirus,” “private problems regarding the corona crisis,” and “fears of the novel coronavirus.” Participants answered on a 7-point Likert scale ranging from “never” to “very often” (T1: *α*=.91; mean 2.30, SD 1.57; T2: *α*=.92; mean 1.87, SD 1.32). Principal component analysis indicated a 1-dimensional scale (eigenvalue=3.12; accounting for 78.00% of the variance).

#### Happiness

We assessed happiness with 3 items derived from Wirth et al [47,48] on a 7-point Likert scale ranging from “never” to “very often.” Participants were asked how often they were “in a good mood,” “happy,” and “joyful” in the last week (T1: *α*=.92, mean 4.86, SD 1.40; T2: *α*=.94, mean 5.20, SD 1.36). Principal component analysis showed that happiness is a 1-dimensional scale (eigenvalue=2.59; accounting for 86.44% of the variance).

#### Psychological Well-being

For psychological well-being, we used 7 items derived from Diener et al [29]. We asked participants to indicate their agreement to the following 7 statements on a 5-point Likert scale ranging from “strongly disagree” to “strongly agree:” “I lead a purposeful and meaningful life,” “I am optimistic about my future,” “My social relationships are supportive and rewarding,” “I am engaged and interested in my daily activities,” “I actively contribute to the happiness and well-being of others,” “I am competent and capable in the activities that are important to me,” and “I am a good person and live a good life” (T1: *α*=.88, mean 3.97, SD 0.72; T2: *α*=.90, mean 3.95, SD 0.77). Principal component analysis resulted in a 1-dimensional scale (eigenvalue=4.05; accounting for 57.85% of variance).

#### Control Variables

As controls, we assessed participants’ age, gender (1=male; 2=female), and education (compulsory school levels=low; secondary school levels=moderate; high school and university levels=high). Additionally, we controlled for the sampling method using a dummy variable (0=polling quota sample data; 1=university quota sample data).

### Statistical Analysis

Using SPSS Amos [49], we conducted structural equation modeling with full information maximum likelihood estimation. Apart from our control variables, we controlled for autoregressive effects (eg, fear of COVID-19 at T1 as a predictor of fear of COVID-19 at T2). Furthermore, we estimated all reciprocal effects.

Before we conducted the data analysis, we additionally checked for longitudinal measurement invariance of all outcome variables by constraining all factor loadings of our latent variables at T1 and T2 [50]. Based on the comparative fit index (CFI), Tucker-Lewis Index (TLI), Bentler-Bonett Normed Fit Index (NFI), chi-square to degrees of freedom ratio (*χ*^2^/df), and root mean square error of approximation (RMSEA), the goodness of fit of the model can be considered as good (CFI=0.95; TLI=0.93; NFI=0.91; *χ*^2^/df=2.26, *P*<.001; RMSEA=0.04; 90% CI 0.04-0.05). We established a partial metric invariance model that does not fit the data worse compared to the unconstrained model (*χ*^2^_9_=16.54, *P*=.06). For this partial metric invariance model, 1 factor loading for happiness and 2 for well-being were not forced to be equal over time. Thus, partial metric invariance over time was established, indicating no substantial differences in meaning of all latent variables over time [50].

## Results

**Figure 2:**
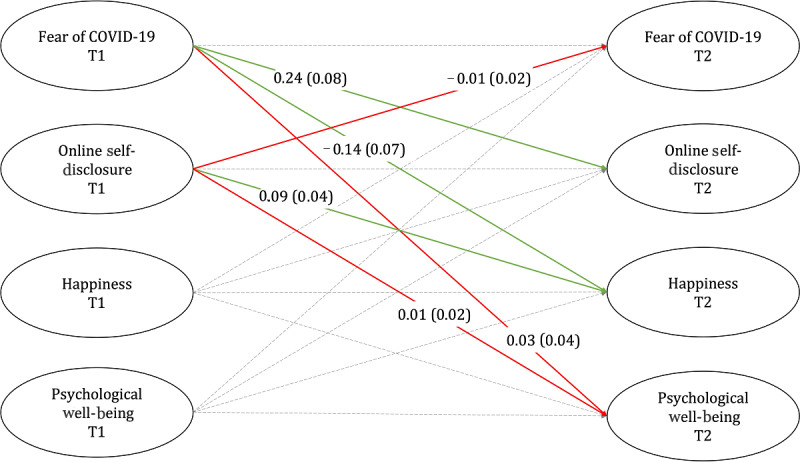
Summary of the principal results of the autoregressive structural equation model. Solid lines indicate the paths (presented with unstandardized path coefficients and standard errors in parentheses) that were included in the hypotheses, and dashed lines indicate the paths that were involved in statistical analysis (for detailed results including covariates, see Multimedia Appendix 1). Significant paths are displayed in green, and nonsignificant paths are displayed in red.

Zero-order correlations and the main results of the structural model are shown in Table S1 and Table S2 in Multimedia Appendix 1. In line with H1, findings from the autoregressive latent variable model suggests that fear of COVID-19 positively predicts online self-disclosure on social media over time (*b*=0.24, SE=0.08, *P*=.003) (Figure 2). Neither happiness nor psychological well-being measured at T1 predicted online self-disclosure on social media at T2. However, we found that higher educated individuals were more likely to self-disclose online over time (*b*=0.30, SE=0.13, *P=*.03).

In H2, we assumed that fear of COVID-19 negatively predicts (1) happiness and (2) psychological well-being over time. While we found support for H2a (*b*=−0.14, SE=0.07, *P=*.04), there was no support for H2b (*b*=0.03, SE=0.04, *P=*.48). Furthermore, there were significant positive relationships between age and psychological well-being (*b*=0.01, SE=0.00, *P=*.004), as well as between female gender and psychological well-being (*b*=0.13, SE=0.06, *P=*.03).

In line with H3a, we found that online self-disclosure on social media positively predicted happiness (*b*=0.09, SE=0.04, *P=*.02). However, against the expectation formulated in H3b, online self-disclosure was found to be unrelated to psychological well-being over time (*b*=−0.01, SE=0.02, *P=*.57). Yet, it is important to note that psychological well-being and happiness were significantly related over time (happiness T1 → psychological well-being T2: *b*=0.11, SE=0.03, *P<*.001; psychological well-being T1 → happiness T2: *b*=0.42, SE=0.09, *P<*.001).

Finally, we found no support for H4, as online self-disclosure was not a significant negative predictor of fear of COVID-19 over time (*b*=−0.01, SE=0.02, *P=*.57). Combined with the findings for our first hypothesis, this suggests that fear can prompt self-disclosure, but the reciprocal relationship cannot be observed. Moreover, fear was not significantly predicted by any other variable in our data set, except for psychological well-being, indicating that individuals with high psychological well-being are less likely to experience fear of COVID-19 over time compared to individuals with lower scores (*b*=−0.15, SE=0.07, *P=*.04).

## Discussion

### Principal Findings

This study provides empirical support for the coping strategy of online self-disclosure. More specifically, our main findings reveal that more intense COVID-19 fears at the beginning of the first COVID-19 lockdown (T1) predicted greater online self-disclosure at the end of the lockdown 1 month later (T2), indicating that it has been considered a relevant means of coping in light of governmental restrictions. Further, the study shows that greater engagement in online self-disclosure at T1 coincides with increased feelings of happiness at T2, suggesting coping effectiveness with regard to hedonic emotional states. Notably, psychological well-being at T2 was not linked to COVID-19 fears and online-self disclosure at T1.

In the time of COVID-19, people all over the world have been confronted with significant psychological stressors, such as the likelihood of being infected or the social isolation resulting from lockdown measures. Social media provide a way to cope with these stressors, especially by disclosing self-related private information to other people. Such online self-disclosure has an important psychological function. By disclosing their concerns, fears, or personal problems during the lockdown, individuals connect with others and maintain the feeling of not being alone, which may save them from harmful health-related consequences.

In this study, using partially metric measurement invariant structural equation modeling, we found the first empirical evidence that fear of COVID-19 increased smartphone-based self-disclosure on social media, which, in turn, fostered individuals’ happiness. Three aspects of our findings are particularly noteworthy. First, while fear of COVID-19 fostered online self-disclosure over time, we did not observe any evidence for the reverse effect. This means fear leads people to disclose their concerns using digital media, but this does not help to alleviate fear. Interestingly, happiness, which was positively predicted by online self-disclosure, did not reduce fear of COVID-19 over time either. In order to interpret these findings, we need to keep in mind that the study was conducted during the first lockdown in Austria in spring 2020, where the uncertainty regarding the pandemic, the epidemiologic characteristics of the coronavirus, and its consequences for human life were arguably at its peak in many countries. In other words, no matter the amount of online self-disclosure, fears of COVID-19 remained present, most likely due to the ongoing risks and uncertainties associated with it. In fact, the only construct that was found to reduce fear over time was psychological well-being. Individuals who report a high degree of psychological well-being may have more confidence in their ability to master or control the virus or exhibit stronger resilience against adverse psychological states [51]. Clearly, more empirical insight is needed to explain this finding.

Second, it is noteworthy that, compared to happiness, we did not observe a relationship between online self-disclosure and psychological well-being. Happiness can be understood as a relatively transient hedonic emotional state in which individuals experience pleasure and joy [29]. By contrast, psychological well-being includes more stable and long-term eudaimonic indicators such as life satisfaction and quality of life [52]. This refers to the notion that our current life and our life achievements are perceived as being close to our general ideals and goals in life [53]. The inconsistent effectiveness of online self-disclosure for coping may be explained by the contrast in both indicators’ scopes. Disclosing to others how one personally feels about COVID-19, even if done repeatedly over the course of 4 weeks, may come as a habitualized temporal relief from pandemic-related stressors; however, it might not by itself be capable of changing the bleak circumstances that created these stressors in the first place (nor stressors unrelated to the pandemic) in such a brief period. Within 4 weeks, online self-disclosure may therefore “only” facilitate ephemeral affective states that got repeatedly threatened by COVID-19 but not more stable long-term self-evaluations that most likely go beyond the circumstances of the pandemic. However, as evident in our data, happiness and psychological well-being are positively and reciprocally related over time. This may suggest that online self-disclosure may impact psychological well-being indirectly, that is, by affecting happiness in the first place, which then influences individuals’ psychological well-being. Unfortunately, with only 2 panel waves, we lack the data to test such mediated relationships.

Notwithstanding this limitation, one could argue that happiness serves as an important psychological resource in times of crisis. In states of happiness, individuals are likely to select and process emotionally congruent (ie, positive) information, which may facilitate self-confidence and prevent the emergence of depressive tendencies [54]. One could also assume that happy individuals differ from unhappy individuals in the ways in which they cope with the flow of negative media information surrounding the COVID-19 pandemic [55]. Of course, additional data are needed to firmly test these conjectures.

Third and finally, online self-disclosure was only predicted by fear and not by happiness and psychological well-being, pointing to the very nature of self-disclosure, that is, self-disclosure can be understood as a means to cope with *negative* stressors and not to communicate *positive* experiences. Accordingly, online self-disclosure is not reduced (nor enhanced) in positive life situations. The combination of these findings supports the notion that online self-disclosure comes as a suitable coping strategy in the face of physical distance and government restrictions impeding face-to-face–based coping that appears to withstand the general positivity bias in social media environments.

### Limitations

Some limitations need to be acknowledged. Lockdowns and other government measures to fight COVID-19 differ between countries, which may have a significant impact on self-disclosure behaviors. Thus, findings from Austria may not be generalizable to other countries, necessitating additional empirical evidence. Related to this, data collection in this study started at the beginning of the lockdown in March/April 2020, lasting until the end of the lockdown in May 2020 in Austria. The findings may thus not be generalizable to the entire period of the COVID-19 pandemic, such as subsequent COVID-19 lockdowns, that is, individuals may behave in different ways during a second lockdown than they did during the first lockdown. Therefore, familiarity with a lockdown situation and compliance with government measures should be taken into account in future research. Moreover, future studies should use more measurements, ideally spanning over lockdown and nonlockdown periods.

With regard to methodology, we relied on participants’ self-reports to measure online self-disclosure. Clearly, self-report data have limitations as they are prone to social desirability and other measurement errors. Participants in this study may have underestimated or overestimated their levels of online self-disclosure. Related to this, our measures and data allow no assumptions about the content of online self-disclosure. Individuals may have enclosed several emotional states, such as fears, practical problems, economic issues, and health-related aspects. In order to shed light on the content of self-disclosure during the pandemic, qualitative methodologies are particularly warranted. Moreover, although we could observe effects over the course of 1 month, our research design did not allow any conclusions about longitudinal mediation mechanisms. Future research should continue this work with more panel waves. Such data would also account for mediation paths among fear of COVID-19, online self-disclosure, and psychological outcomes.

Finally, the relationship between online self-disclosure and psychological well-being may depend on individual predispositions. For instance, chronically distressed individuals may not benefit from online self-disclosure [32]. The reason can be found in their distress-related tendency to share more negative or inauthentic information, which is usually accompanied by more negative received feedback. Future research should investigate these individual-level contingent conditions.

### Conclusions

Taken together, our findings suggest that self-disclosure in online digital media can play a major role in restoring people’s happiness during COVID-19 and the lockdowns it has brought upon us. With the worldwide spread of COVID-19, fear was a prevalent emotional reaction among many people. To deal with this fear, individuals used digital media, especially social media via their smartphones, to disclose their feelings, problems, and concerns. We showed that such online self-disclosure opportunities contributed to the psychological health of individuals. Although online self-disclosure did not affect psychological well-being, it made people happier over time, which is an important psychological resource in times of uncertainty, crisis, and stress.

## Appendix

Multimedia Appendix 1 Zero-order correlations and results of the structural equation model.

## Abbreviations

CFI: comparative fit index
NFI: Bentler-Bonett Normed Fit Index
RMSEA: root mean square error of approximation
TLI: Tucker-Lewis Index
