# Supplementary material for: Longitudinal Relationships Among Fear of COVID-19, Smartphone Online Self-Disclosure, Happiness, and Psychological Well-being: Survey Study
Source: J Med Internet Res. 2021 Sep 27;23(9):e28700. doi: 10.2196/28700 (PMC8477910; doi:10.2196/28700)
Supplement: Multimedia Appendix 1 [file jmir_v23i9e28700_app1.docx]

**Multimedia Appendix 1.** Zero-order correlations and results of the structural equation model.

**Table S1.** Zero-order correlations.

| Variables^a^ | 1 | 2 | 3 | 4 | 5 | 6 | 7. | 8 |
| --- | --- | --- | --- | --- | --- | --- | --- | --- |
| 1. Fear of COVID-19 (T1) | 1 |  |  |  |  |  |  |  |
| 1. Fear of COVID-19 (T2) | .79  (*P* < .001) | 1 |  |  |  |  |  |  |
| 1. Online Self-Disclosure (T1) | .40  (< .001) | .35  (< .001) | 1 |  |  |  |  |  |
| 1. Online Self-Disclosure (T2) | .35  (< .001) | .44  (< .001) | .47  (< .001) | 1 |  |  |  |  |
| 1. Happiness (T1) | -.20  (< .001) | -.21  (< .001) | -.11  (.005) | -.12  (.012) | 1 |  |  |  |
| 1. Happiness (T2) | -.18  (< .001) | -.24  (< .001) | -.01  (.823) | -.26  (< .001) | 0.65  (< .001) | 1 |  |  |
| 1. Psychological Well-Being (T1) | -.09  (.043) | -.18  (< .001) | -.06  (.179) | -.13  (.013) | .50  (< .001) | .51  (< .001) | 1 |  |
| 1. Psychological Well-Being (T2) | -.04  (.404) | -.20  (< .001) | -.06  (.216) | -.23  (< .001) | .47  (< .001) | .61  (< .001) | .70  (< .001) | 1 |

*^a^N*_T1_ = 731, *N*_T2_ = 416; T1 = Time 1, T2 = Time 2.

**Table S2.** Results of the structural equation model.

| Predictor^a^ | Fear of COVID-19  (T2) | | | | Online Self-Disclosure  (T2) | | | | Happiness  (T2) | | | | |  | Psychological Well-Being  (T2) | | | |
| --- | --- | --- | --- | --- | --- | --- | --- | --- | --- | --- | --- | --- | --- | --- | --- | --- | --- | --- |
|  | | | *B* | *SE* | | | *B* | *SE* | | *B* | | *SE* | | | | *b* | *SE* | |
| Age | | .00 (*p* = .988) | | .00 | | | -.00 (.530) | .00 | | .01 (.218) | | .00 | | | | .01 (.002) | .01 | |
| Gender (women) | | -.04 (.643) | | .09 | | | -.09 (.447) | .12 | | .10 (.340) | | .11 | | | | .13 (.034) | .06 | |
| Education (low) | | -.05 (.634) | | .11 | | | .20 (.185) | .15 | | .09 (.519) | | .14 | | | | -.02 (.803) | .08 | |
| Education (high) | | .03 (.783) | | .10 | | | .30 (.025) | .13 | | -.10 (.402) | | .12 | | | | .09 (.196) | .07 | |
| Sample type | | -.03 (.733) | | .09 | | | -.44 (< .001) | .13 | | .11 (.325) | | .11 | | | | .12 (.060) | .07 | |
| Fear of COVID-19 (T1) | | .83 (< .001) | | .07 | | | .24 (.003) | .08 | | -.14 (.043) | | .07 | | | | .03 (.479) | .04 | |
| Online Self-Disclosure (T1) | | .03 (.346) | | .03 | | | .33 (< .001) | .04 | | .09 (.022) | | .04 | | | | -.01(.574) | .02 | |
| Happiness (T1) | | .00 (.964) | | .04 | | | .00 (.982) | .06 | | .56 (< .001) | | .05 | | | | .11(< .001) | .03 | |
| Psychological Well-Being (T1) | | -.15 (.040) | | .07 | | | -.09 (.353) | .10 | | .42 (< .001) | | .09 | | | | .60 (< .001) | .06 | |
| R^2^ | | .64 | | | | .30 | | | | | .48 | |  | | | .54 |  |  |

*^a^N*_T1_ = 731, *N*_T2_ = 416; T1 = Time 1, T2 = Time 2.
